# Opportunities for Research in Mental Health Emergencies: Executive Summary and Methodology

**DOI:** 10.5811/westjem.2019.1.39260

**Published:** 2019-02-19

**Authors:** Michael P. Wilson, Christina Shenvi, Loren Rives, Kimberly Nordstrom, Sandra Schneider, Michael Gerardi

**Affiliations:** * University of Arkansas for Medical Sciences, Department of Emergency Medicine, Little Rock, Arkansas; † University of North Carolina, Department of Emergency Medicine, Chapel Hill, North Carolina; ‡ American College of Emergency Physicians, Irving, Texas; § University of Colorado School of Medicine, Department of Psychiatry, Denver, Colorado; ¶ John Peter Smith Hospital, Department of Emergency Medicine, Fort Worth, Texas; || Hofstra Northwell School of Medicine, Hempstead, New York; # Morristown Memorial Hospital, Morristown, New Jersey

## Abstract

**Introduction:**

Despite the ever-increasing numbers of mental health patients presenting to United States emergency departments, there are large gaps in knowledge about acute care of the behavioral health patient. To address this important problem, the Coalition on Psychiatric Emergencies convened a research consensus conference in December 2016 consisting of clinical researchers, clinicians from emergency medicine, psychiatry and psychology, and representatives from governmental agencies and patient advocacy groups.

**Methods:**

Participants used a standardized methodology to select and rank research questions in the order of importance to both researchers and patients.

**Results:**

Three working groups (geriatrics, substance use disorders, and psychosis) reached consensus on 26 questions within their respective domains. These questions are summarized in this document.

**Conclusion:**

The research consensus conference is the first of its kind to include non-clinicians in helping identify knowledge gaps in behavioral emergencies. It is hoped that these questions will prove useful to prioritize future research within the specialty.

## INTRODUCTION

Emergency departments (ED) across the country are increasingly a point of care for patients with acute mental and behavioral health needs. From 1992–2001, approximately 53 million visits to United States (U.S.) EDs were due primarily to mental health concerns.^1^ Patients often present during an acute mental health crisis, with suicidal ideation, homicidal ideation, agitation, substance abuse or withdrawal, acute psychosis, or following a suicide attempt.^2^–^5^ The assessment of patients with behavioral health needs is challenging in part because of the varied nature of the presentations, the frequent coexistence of medical and psychiatric disorders, and the difficulty in obtaining a reliable history and exam from patients who may be uncooperative, intoxicated, have major neurocognitive disorders, or be delirious. Furthermore, assessment and treatment in the ED can be challenging due to insufficient space, time, staff, and resources. To help provide leadership and improvements in emergency mental health care in U.S. EDs, the Coalition on Psychiatric Emergencies (CPE) was founded to promote education, policies, and research that will ultimately improve the quality of behavioral healthcare for patients.

Since the 1994 Macy report, promotion of research within emergency medicine (EM) has had a number of successes, including the successful establishment of the Emergency Medicine Foundation.^6^–^8^ Promotion of research and training in behavioral emergencies, however, has not garnered the same level of attention. Training in behavioral emergencies was almost non-existent outside of psychiatry before the 19th century,^9^ and the subspecialty of emergency psychiatry was not established until 1988. Prior to that time, individuals with mental illness, when they were treated by physicians at all, were treated by general practitioners with little formal training. The development of specialties in medicine led to the establishment of board certification and mandated lifelong learning, which in other areas of medicine has been associated with improved outcomes for patients.^10^

Given that EDs now provide the majority of care for patients who are admitted,^11^ the ED has naturally served a similar function for behavioral health patients as well.^12^ Given the open access and availability of EDs nationwide, they are also frequently the only source of care for mental health patients who may have poor healthcare literacy, inadequate access to care, or insufficient insurance.^13^–^14^ Thus, contemporary EDs are uniquely positioned to address acute behavioral emergencies.^3^ Unfortunately, despite the importance of EDs in caring for mental health patients, there are currently many gaps in our understanding of optimal ED care for behavioral emergencies. Although the U.S. leads the world in EM research, only a small proportion of this research is dedicated to psychiatric emergencies.^15^–^16^ Thus, there remains great need for further prioritization, collaboration, and investment in this area.

To address this need, the CPE convened a research consensus conference with experts from both psychiatry and EM. Unlike previous consensus conferences in this area, which included only clinicians and/or research scientists,^1^, ^17^ the current conference instead convened a diverse set of organizations representing clinician stakeholders, clinical researchers, psychology, governmental agencies and patients’ advocacy organizations so as to ensure that resulting priorities reflected both patient priorities and scientific need.^2^, ^3^, ^12^, ^18^–^23^

### Objectives

The objectives of the conference were to highlight and prioritize areas of greatest research need within selected domains of emergency psychiatry while taking the patient perspective into account, and then to summarize these recommendations into consensus documents.

## METHODS

### The Coalition on Psychiatric Emergencies

CPE includes over a dozen professional organizations, patient advocacy groups, and systems of care,^24^ all with an interest in behavioral emergencies. The steering committee at the time of the conference consisted of representatives from the following organizations: the American College of Emergency Physicians (ACEP), the American Association for Emergency Psychiatry, the Depression and Bipolar Support Alliance, the National Alliance on Mental Illness, and the Emergency Nurses Association. The steering committee was responsible for identification of priority domains, planning the conference, inviting participants and stakeholders, and determining the methodology.

### Conference Methodology

This structured expert consensus conference was held December 7, 2016. The overarching question of the conference was to investigate whether early treatment might positively affect outcomes for patients with mental health crises,^25^ similar to other critical conditions of the conference.^25^ By consensus, the CPE steering committee identified four priority domains on which to focus: geriatric behavioral health emergencies; suicidality and acute depression; substance use disorders (SUD); and acute psychosis. As in previous conferences of these types, the four domains were chosen a priori based on their importance to providers currently caring for patients with behavioral emergencies.^17^

The 35 participants in the conference were sorted into working groups by self-identified interest and expertise. While participants each worked in a single group, they were able to provide feedback and comments on the priorities identified by other working groups both during the conference and after. Each workgroup appointed a moderator who conducted the consensus building during the conference (see below), and a group leader who identified relevant articles prior to meeting in person. Each participant was provided with these articles and was free to contribute any additional articles desired.

Consensus building on research questions within each domain was accomplished by use of the nominal group technique in person.^26^–^27^ The nominal group technique is a four-step process in which participants are invited to identify ideas and raise exploratory questions, record these ideas, discuss them freely, iteratively focus and revise them, and then vote on relative importance. Participants work independently but in the presence of one another. This method was chosen as it has the advantage of preventing any particular expert from dominating the conversation or influencing the voting.

Specific research ideas, questions, and question variants were voted on in person using the dot method. Questions that received more votes were deemed to be more important, and thus were ranked more highly within each domain. As research on behavioral emergency questions are of importance to industry, representatives of pharmaceutical companies were permitted to attend. However, those representatives were not allowed to vote on the final wording or rank order importance of any question.

At the end of the conference day, all groups presented their research questions to all stakeholders. Stakeholders from other priority domains were permitted to ask questions or make clarifying points, but were not permitted to vote on any research question. After the conference, each group was allowed to form additional consensus on the final form of each question in any manner desired, typically by email. However, stakeholders from other priority domains were not permitted further opportunities to revise or edit these questions.

### Identification of Relevant Stakeholders

Identification of relevant stakeholders (i.e., conference participants) was accomplished primarily by a web search for publications in each particular domain. The search strategy was not conducted with formalized keywords, but identified stakeholders were expected to have either one or more publications in the relevant domain or have given lectures in this area at a national conference. As this method of identification would be expected to weight the participant list most heavily towards researchers, clinicians with relevant interest and expertise were also identified by member organizations on the CPE steering committee. Individuals representing patient advocacy groups and governmental agencies, also nominated by member organizations on the CPE steering committee, were included in order to create a robust and diverse set of expert opinions. The inclusion of non-clinicians and non-clinical researchers was an important difference between this conference and previous conferences of this type.^1^, ^17^ Expert participants were asked to self-declare conflicts of interest using the standard ACEP conflict-of-interest form for committees.

## RESULTS

A total of 35 stakeholders (57% female, average age 47), including 13 non-clinicians, with an average of 17 years in their relevant fields participated in the consensus conference. The research priorities identified by each working group are listed more fully in the accompanying articles. However, the following themes emerged from the conference and had consensus from both the participants in each group and non-group stakeholders. With regard to geriatrics, more research is needed on identification, screening, and management of older adults at risk for worse outcomes because of behavioral emergencies.^28^ This includes, but is not limited to, appropriate SUD screening for older adults specifically as an important component of the medical screening exam.

With regard to substance use, more research is needed on screening and intervention for substance use in the ED.^29^ Indeed, in the period of time that has elapsed since this conference the director of the National Institute of Drug Abuse has made public comments likening the failure of EDs to provide treatment options to patients with SUD as potential “malpractice.”^30^ In the psychosis workgroup, better methods for screening, measurement, and evaluation of psychosis are needed. More patient involvement is needed to determine the most relevant patient outcomes for emergency treatment of psychosis.^31^

Despite initial agreement on some important questions by the suicide workgroup during the conference, this working group was unable to agree on the final format of research questions after the conference. Consequently, although the expert participants recognize the importance of suicide-related research to both patients and families, no key research questions are available from this group.

## DISCUSSION

Summaries of the most important recommendations from the working groups are outlined below.

- Tables 1 – 3: Research questions regarding older adults with behavioral changes.
- Table 4: Research questions regarding individuals with SUD and behavioral emergencies.
- Tables 5 – 6: Research questions regarding individuals with acute psychosis.

## CONCLUSION

More research is needed in the area of acute mental and behavioral health disorders in order to care for patients in the acute care setting more effectively. This research consensus conference, organized by the multi-disciplinary Coalition on Psychiatric Emergencies, was the first of its kind to build consensus from a group with diverse expertise and experience to prioritize research goals in four domains within mental health and behavioral emergencies. Continued consensus building among diverse stakeholders in this field should be an ongoing priority, as research in these domains has implications for both practicing medical personnel and the individuals in their care.

## Figures and Tables

**Table 1:** Key research questions to guide efforts for improved care of older adults with behavioral changes through screening and identification.

Question 1	What are the barriers to screening for alcohol or substance use in older adults?
Question 2	Using age as a stratification method, what are the medical and radiographic components of an appropriate medical screen for patients with psychiatric symptoms with an emphasis on sensitivity, specificity, and accuracy; do routine screening labs, including urine, affect management and disposition in older adults with psychiatric symptoms?
Question 3	How often does noncompliance with prescribed medications contribute to emergency department presentations with agitation or behavioral changes?

**Table 2:** Key research questions to guide efforts for improved care of older adults with behavioral changes through improved management strategies.

Question 4	What is the most effective pharmacologic agent to manage acute agitation in the acute care setting?
Question 5	Does earlier treatment with psychotropic medications decrease length of stay in the emergency department (ED) for elderly agitated patients and does choice of treatment matter?
Question 6	How often are older adults restrained physically or chemically in the ED; does the rate of restraint use vary with underlying psychiatric disorders, and what are the harms or benefits of their use?
Question 7	What are barriers to initiating pharmacologic treatment for acute psychiatric illness in the ED among older adults?
Question 8	Does the initiation of home-based services for patients discharged from the ED with dementia help reduce the rate of ED return visits?
Question 9	What are the necessary components of an effective decision-support tool to determine whether it is safe to start or stop psychiatric medications, and does the use of such a tool improve outcomes?

**Table 3:** Key research questions to guide efforts for improved care of older adults with behavioral changes through improved identification and management of delirium.

Question 10	What are the barriers to diagnosis of delirium in the emergency department (ED), and how can they be overcome?
Question 11	Is ED length of stay an independent risk factor for the development of delirium?
Question 12	Does ED length of stay contribute to worse morbidity and mortality or adverse medical events in older adults with delirium?
Question 13	What are the most effective non-pharmacologic interventions in the ED to manage or prevent delirium?
Question 14	Does having an ED pharmacist involved in patient care help reduce rates of delirium in the ED?

**Table 4:** Key research questions to guide emergency department-based interventions for substance use disorders.

Question 1	What are the most effective, efficient and appropriate ways to screen for SUD in the ED?
Question 2	What are the most effective ED-based interventions for SUDs?
Question 3	What is the role for initiation and management of SUD treatment and detoxification in the ED?
Question 4	What is the role of sociocultural and generational factors in acceptability, accessibility, and benefit of ED-based initiatives?
Question 5	What are the best practices for the evaluation and management of the acutely intoxicated patient?
Question 6	What role can peer mentors, or patient navigators, play in improving patient outcomes?

*ED,* emergency department; *SUD*, substance use disorder.

**Table 5:** Key research questions to guide efforts for individuals with psychosis through screening and identification.

Question 1	Can a research-based triage tool be developed to assess psychosis in emergency department patients?
Question 2	What outcomes are meaningful for patients/families when assessing the effectiveness of psychosis interventions?

**Table 6:** Key research questions to guide efforts for effective interventions and management of the patient with acute psychosis.

Question 3	What is the recommended treatment for psychosis in the emergency setting?
Question 4	What affects emergency provider decision-making in treatment choice for psychosis?
Question 5	What system outcomes can be affected by early treatment of psychosis in emergency settings - both within the emergency care setting and thereafter?
Question 6	Are there appropriate care locations for psychotic patient presentations instead of the emergency department?
